# Influence of type 2 diabetes on symbolic analysis and complexity of heart rate variability in men

**DOI:** 10.1186/1758-5996-6-13

**Published:** 2014-02-01

**Authors:** Sílvia CG Moura-Tonello, Anielle CM Takahashi, Cristina O Francisco, Sérgio LB Lopes, Adriano M Del Vale, Audrey Borghi-Silva, Angela MO Leal, Nicola Montano, Alberto Porta, Aparecida M Catai

**Affiliations:** 1 Physiotherapy Department, Cardiovascular Physiotherapy Laboratory, Nucleus of Research in Physical Exercise, Federal University of São Carlos, São Paulo, Brazil; 2 Department of Medicine, Federal University of São Carlos, São Paulo, Brazil; 3 Department of Clinical Sciences, Internal Medicine II, L. Sacco Hospital, University of Milan, Milan, Italy; 4 Department of Technologies for Health, Galeazzi Orthopaedic Institute, University of Milan, Milan, Italy

**Keywords:** Heart rate variability, Type 2 diabetes mellitus, Symbolic analysis, Shannon entropy and conditional entropy

## Abstract

**Background:**

Individuals with diabetes may develop cardiac autonomic dysfunction that may be evaluated by heart rate variability (HRV). The aim was evaluated heart rate variability (HRV) of individuals with type 2 diabetes, without cardiovascular autonomic neuropathy (CAN), in response to active postural maneuver by means of nonlinear analysis (symbolic analysis, Shannon and conditional entropy) and correlate HRV parameters between them, glycated hemoglobin and diabetes duration.

**Methods:**

Nineteen men with type 2 diabetes without CAN (T2D) and nineteen healthy men (CG), age-range from 40 to 60 years were studied. We assessed HRV in supine and orthostatic position using symbolic analysis (0V%, 1V%, 2LV% and 2UV%), Shannon and conditional entropy (SE and NCI).

**Results:**

In supine position T2D presented higher sympathetic modulation (0V%) than CG. However, there was not any difference between groups for indexes of complexity (SE and NCI). Furthermore, T2D presented a preserved response of cardiac autonomic modulation after active postural maneuver.

**Conclusions:**

The present study showed that individuals with type 2 diabetes without CAN presented higher cardiac sympathetic modulation. However, the complexity of HRV was not influenced by imbalance of the autonomic modulation in individuals with type 2 diabetes. In addition, the response of autonomic nervous system in the heart remains preserved after active postural maneuver in individuals with type 2 diabetes, possibly due to the lack of CAN in this group.

## Background

Type 2 diabetes is a metabolic disorder of multiple etiologies characterized by hyperglycemia, insulin resistance and a variable degree of insulin secretory deficiency [1,2]. It is considered as a cardiovascular risk factor, as individuals with diabetes have double the risk of developing acute myocardial infarction and stroke than those who do not have it [3].

Individuals with diabetes may develop autonomic dysfunction related to the cardiovascular system, known as cardiovascular autonomic neuropathy (CAN). This dysfunction occurs where there are lesions in the peripheral autonomic fibers [4].

Cardiac autonomic dysfunction, characterized by alterations in the modulation the cardiac vagal, or sympathetic efferents, or in both, may be evaluated by heart rate variability (HRV) [5], which is a simple, non invasive measurement [6,7] and is considered a predictor of cardiovascular morbidity and mortality [8]. It can be evaluated under conditions of rest [9,10], and during provocative tests such as physical exercise [11,12], the tilt test [13,14] and active postural maneuver [15,16].

Postural change promotes stimulus of the autonomic nervous system (ANS) in the heart by inducing an increase in sympathetic modulation during the tilt test [14,17] and with active postural maneuver [16,18]. In addition, active postural maneuver is a simpler evaluation when compared with the tilt test*,* and it is, also, a low cost method that can be performed at the bedside [16,18].

The HRV may be evaluated by linear and nonlinear methods. Javorka et al. [9] developed study involving nonlinear analysis to assess the complexity of HRV (Shannon and conditional entropy) of young patients with type 1 diabetes. They observed reduction in the complexity of HRV for this population. In addition, Javorka et al. [9] performed symbolic analysis and observed reduction index 2LV% (index that reflects parasympathetic and sympathetic modulation with parasympathetic predominance). The authors attributed these results to vagal dysfunction [9]. However, there is a scarcity of studies that evaluate the HRV by nonlinear analysis in patients with type 2 diabetes.

Therefore, the primary objective was to evaluate the HRV of individuals with type 2 diabetes, without cardiovascular autonomic neuropathy (CAN), in response to active postural maneuver from the supine to orthostatic position, by means of nonlinear analysis (symbolic analysis, Shannon and conditional entropy). Our secondary aim was to correlate the duration of diabetes and glycated hemoglobin (HbA1c) with used HRV indexes.

## Methods

### Subjects

Thirty eight male volunteers, age-range from 40 to 65 years were evaluated. They were divided into two groups: one of subjects with type 2 diabetes in accordance with the recommendations of the American Diabetes Association [19] (T2D; n = 19) and the other group of control subjects (Control Group – CG; n = 19).

Volunteers were selected in accordance with the following criteria: sedentary, with aerobic functional classified as: very weak, weak, or reasonable according to the American Heart Association [20], non-smokers, non-drinkers and people without any kind of lung disease or systemic inflammatory disease. The exclusion criteria were as follows: diagnostic CAN (verified by clinical evaluation by an endocrinologist, slow deep breathing autonomic testing – according to O’Brien et al. [21]; tachycardia at rest – heart rate > 100 bpm; orthostatic hypotension in reaction to active postural change – systolic blood pressure > 30 mmHg) [21], myocardial ischemia and/or cardiovascular pathologies on clinical examinations and clinical ergometric test conducted by a Physician, disease that incapacity the subject to remain in the orthostatic position actively, or walk and/or pedal the bicycle, and a body mass index (BMI) greater than 29.99 Kg/m^2^ for the CG.

The present study was approved by the Ethics Committee on Research in Human Beings of the Federal University of São Carlos (Protocol N. 093/2011). All the subjects who participated in the study were informed about the experimental procedures and signed a Formal Consent Agreement.

### Experimental procedure

The experimental procedures were performed in the Cardiovascular Physiotherapy Laboratory at the Federal University of São Carlos and only blood collection was performed at the Clinical Analysis Laboratory. The study was always conducted in the morning period, considering the effects of the circadian rhythm. The experiments were carried out in a climate-controlled room (22-23°C) with a relative air humidity of 50-60%.

The protocols were performed in two days with a difference of one week between them: 1) blood collection, body composition evaluation, autonomic testing and cardiopulmonary exercise testing; 2) instantaneous R–R intervals (RRi) were recorded for HRV analysis. The volunteers were instructed not to practice moderate or heavy exercise, and not ingest food and/or stimulating or alcoholic beverages within 24 hours before the evaluations, and to fast for 12 hours on the day of blood collection.

### Laboratory exams

The volunteers were subjected to venous blood collection for lipid profile, C-reactive protein (CRP), glycated hemoglobin (HbA1c) analysis, and afterwards the mean estimated glycemia was calculated. For these analyses ADVIA 1800 Chemistry System (Siemens, Tarrytown, NY, USA) was used.

### Body composition

The body composition monitor Tanita Ironman (Tanita Corporation of America Inc, Illinois, USA) was used to evaluate the total body fat percentage of the volunteers.

### Cardiopulmonary exercise testing

The symptom-limited cardiopulmonary exercise testing [22], was performed to confirm that subjects were classified at the same functional class [20]. The maximum volume of O_2_ consumption (VO_2_ peak) was determined during an incremental cycle ergometer exercise, with increments calculated by the Wasserman formula [23], using a metabolic analyzer (CPX/D, MedGraphics, St. Paul, MN, USA).

### Heart rate variability (HRV)

The HRV was obtained by the cardiofrequencymeter Polar® RS800CX™ (Polar Electro Oy, Kempele, Finland). This system captures the R wave of the ECG, with a sampling frequency of 500 Hz, thus calculating the HR instantly and storing the RRi in the same way it is done with the Polar® S810i cardiofrequencymeter described by Vanderlei et al. [24].

The volunteers were instructed to lie down in the supine position, not to move and/or talk during RRi capture. Before starting the volunteers remained at rest in the supine position for 10 min, in order to stabilize the cardiovascular variables. The RRi records the protocol consisted of 10 min, with the volunteer lying on a stretcher. Then the systemic blood pressure was measured. Next, the volunteer was instructed to perform active postural change to the orthostatic position, and the system blood pressure was measured again. Finally, the RRi was recorded for the last time in the orthostatic position for the next 10 minutes.

The series length N was fixed at 256 beats in each position. The sequence of RRi with the greatest stability in the central region of the tachogram was selected for each volunteer in both positions. The same sequence selected was used to perform all the analyses.

### Symbolic dynamics

#### Shannon entropy

The Shannon Entropy is obtained by quantizing of the RRi series into six levels ranging from 0 to 5, that means that each heartbeat corresponds to a symbol according to the level of the heartbeat. Therefore, they are organized into patterns with 3 symbols. The shape and distribution of these patterns was calculated with Shannon entropy (SE). The SE is large if its distribution is flat (all patterns are identically distributed and the series carries the maximum amount of information). However, if there is a subset of more probable patterns, while others are missing or infrequent (e.g., in a Gaussian distribution), SE will be small [25].

#### Symbolic analysis

Symbolic analysis was carried out by grouping the patterns with 3 symbols into four families as follows: (a) no variation (0V: all the symbols are equal, i.e. 2,2,2 or 4,4,4); (b) one variation (1V: 2 consecutive symbols are equal and the remaining symbol is different, i.e. 4,2,2 or 4,4,3); (c) two like variations (2LV: the 3 symbols form an ascending or descending ramp, i.e. 5,4,2 or 1,3,4); and (d) two unlike variations (2UV: the three symbols form a peak or a valley, i.e. 4,1,2 or 3,5,3). The rate of occurrence for each pattern is defined as 0V%, 1V%, 2LV%, and 2UV% [26]. It has been observed that 0V% reflects only sympathetic modulation, 1V% reflects sympathetic and parasympathetic modulation, 2LV% reflects sympathetic and parasympathetic modulation with vagal predominance and 2UV% reflects, exclusively, vagal modulation [14,26].

#### Conditional entropy

According to Porta et al. [26], conditional entropy (CE) measures the amount of information carried by the most recent sample of patterns that cannot be derived from a sequence of values of length of past patterns. CE is assessed with the complexity index (CI). We normalized this index with the Shannon entropy of the RRi to obtain a normalized complexity index (NCI) that expresses complexity in terms of dimensionless units. This index ranges from 0 (null information) to 1 (maximum information). The larger both indexes are, the greater the complexity, the lower the regularity.

### Statistical analysis

The Shapiro-Wilk normality test was used to identify the distribution of data. Non-normal distribution data were transformed by the log10 and they became normal. Afterwards two-way ANOVA was used to analyze variables considering the effect of the group, effect of position and interaction among them. The Holm-Sidak post hoc was used to show the differences. The correlation between duration of diabetes and glycated hemoglobin (HbA1c) with HRV indexes were determined by Pearson. P value < 0.05 was considered statistically significant. The data were presented as mean ± SD. Sigma Plot software for Windows version 11.00 was used for data analysis.

## Results

The data shown in Table 1 were presented to characterize the studied sample. There was significant difference between the groups for HbA1c and estimated mean glycemia, an expected result, since these exams are generally shown to be altered in individuals with diabetes (Table 1) [27].

**Table 1:** Characteristics of evaluated groups

**Characteristics**	**T2D**	**CG**	***P-value***
Age (years)	50.53 ± 6.96	50.26 ± 7.96	0.345
Body mass (Kg)	86.64 ± 13.86	80.24 ± 9.38	0.057
Height (m)	1.74 ± 0.09	1.75 ± 0.08	0.823
BMI (Kg/m^2^)	28.26 ± 4.13	26.21 ± 1.77	0.062
Percentage of total body fat	24.94 ± 6.20	23.38 ± 3.61	0.384
VO2 peak (ml/min)	1672.05 ± 345.01	1850.89 ± 238.09	0.070
Time of diabetes (years)	11.13 ± 6.41	-	-
*Laboratory Exams*			
PCR (mg/dL)	1.12 ± 1.48	0.69 ± 0.66	0.874
HbA1c (%)	8.54 ± 2.15	5.85 ± 0.30	<0.001*
HbA1c (mmol/mol)	69.80 ± 23.55	40.44 ± 3.26	<0.001*
Estimated mean glycemia (mg/dL)	197.79 ± 61.91	119.47 ± 9.95	<0.001*
Total cholesterol (mg/dL)	193.79 ± 28.61	198.21 ± 39.33	0.635
HDL-cholesterol (mg/dL)	45.37 ± 10.32	46.79 ± 11.02	0.573
LDL-cholesterol (mg/dL)	113.53 ± 32.12	119.26 ± 30.62	0.253
VLDL-cholesterol (mg/dL)	33.84 ± 18.49	31.05 ± 16.11	0.452
Triglycerides (mg/dL)	170.47 ± 92.57	156.37 ± 80.06	0.093
*Risk factors*			
Hypertension	2 (10.53%)	-	-
Obesity	5 (26.31%)	-	-
Dyslipidemia	7 (36.84%)	9 (47.37%)	-
*Medications*			
Oral hypoglycemic drugs	8 (42.11%)	-	-
Insulin	4 (21.05%)	-	-
Oral hypoglycemic drugs + insulin	4 (21.05%)		
Anti-hypertensive drugs	5 (26.31%)	-	-
ACE Inhibitor	1 (5.26%)	-	-
- Calcium channel blocker (amlodipina)	1 (5.26%)	-	-
- Angiotensin II receptor antagonist	2 (10.53%)	-	-
- Hydrochlorothiazide	1 (5.26%)	-	-
- Clonidine	1 (5.26%)	-	-
- Hypolipidemic drug	2 (10.53%)	3 (15.79%)	
*Deep breathing testing*			
E/I	1.27 ± 0.20	1.29 ± 0.16	0.352
ΔIE	18.53 ± 14.94	16.73 ± 8.82	0.494

The data are presented in mean ± standard deviation. T2D = group with type 2 diabetes; CG = Control Group; BMI = body mass index; VO_2_ peak = oxygen consumption at peak of physical effort; PCR = C-reactive protein; HbA1c = glycated hemoglobin.*p < 0.05.

T2D group was evaluated by an endocrinologist and was subjected to tests that assess autonomic nervous system (slow deep breathing autonomic testing, tachycardia at rest and orthostatic hypotension in reaction to active postural). All subjects had normal values for autonomic testing and in clinical evaluation all subjects denied dizziness or sweating with postural maneuver, and they did not report sexual dysfunction, sensory loss of vibration, pressure, pain and temperature perception in the lower limbs, therefore all subjects were diagnosed without CAN.

T2D medication to control diabetes (hypoglycemic drugs and/or insulin). In addition, some individuals with diabetes used too antihypertensive, hypolipidemic drugs (Table 1).

Both groups showed an increase in HR and reduction in mean RRi in active postural maneuver. To variance of RRi the groups were different regardless of posture, with T2D presenting lower values than CG in both positions (Table 2).

**Table 2:** Systolic and diastolic blood pressure (mmHg), heart rate (bpm), R-R interval (RRi) and heart rate variance (VAR)

**Variables**	**T2D**				**CG**				***P-value***		
	**Supine**		**Orthostatic**		**Supine**		**Orthostatic**		**Group**	**Position**	**Interaction**
	**Mean ± SD**	**CI**	**Mean ± SD**	**CI**	**Mean ± SD**	**CI**	**Mean ± SD**	**CI**			
SBP (mmHg)	135 ± 16.4	(126.2–143.8)	128.8 ± 16.0	(120.2–137.3)	129.0 ± 9	(124.3–133.9)	122.2 ± 11.00	(116.4–128.0)	0.068	0.056	0.926
DBP (mmHg)	86.3 ± 12.0	(79.8–92.7)	82.8 ± 11.0	(77–88.7)	82.2 ± 8	(78–86.4)	76.4 ± 20.3	(65.6–87.3)	0.131	0.182	0.735
HR (bpm)	72.8 ± 8.7	(68.6–77.0)	83.3 ±11.5	(77.7–88.8)	67.3 ± 10.4	(62.3–72.3)	81.1 ± 13.5	(74.6–87.6)	0.140	<0.001*	0.517
RRi mean (ms)	836.4 ± 106.1	(785.3–887.6)	734.4 ± 107.5	(682.5–786.2)	911.3 ± 135.3	(846.1–976.6)	760.1 ± 131.7	(696.7–823.6)	0.074	<0.001*	0.378
VAR (ms^2^)	485.4 ± 304.3	(338.8–632.1)	327.0 ± 197.1	(232.0–422.0)	1010.6 ± 745	(651.6–1369.7)	763.9 ± 623.6	(463.3–1064.4)	<0.001*	0.187	0.885

The data are presented in mean ± standard deviation (Mean ± SD) and confidence intervals of mean (CI). T2D = group type 2 diabetes; CG = Control Group; SBP = systolic blood pressure; DBP = diastolic blood pressure; HR = heart rate; iRR = R-R interval between R waves; VAR = variance of RRi; *p < 0.05.

**Figure 1:**
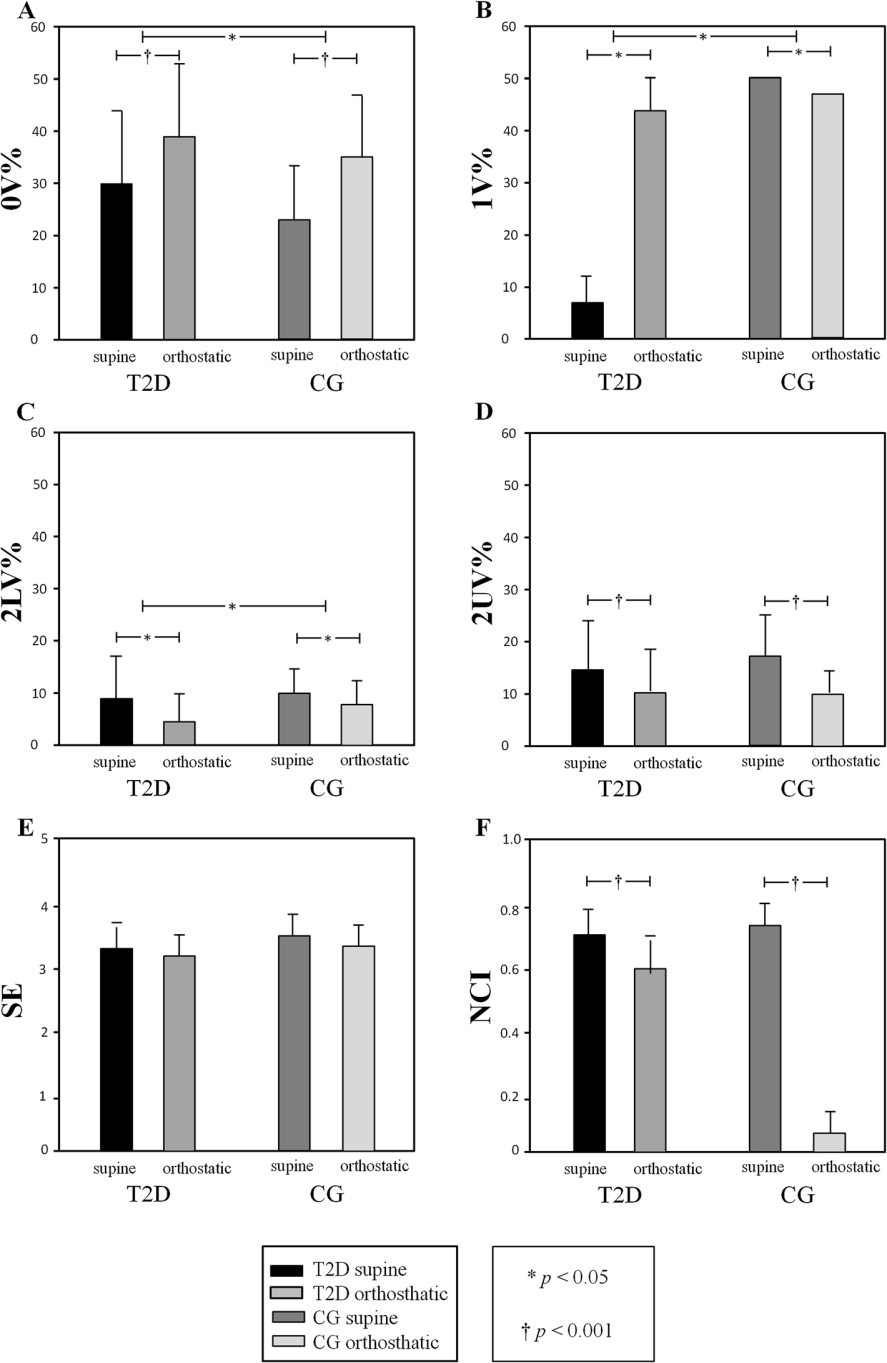
**Symbolic dynamics of HRV for individuals with type 2 diabetes (T2D) and and control group (CG).** The symbol *indicates *p <*0.05 and † indicates *p <*0.001. **A**: 0V% index; **B**: 1V% index; **C**: 2LV% index; **D**: 2UV% index; **E**: SE = Shannon entropy; **F**: NCI = normalized complexity index.

With respect to Shannon entropy, there were not group and position effect. Whereas the normalized complexity index (NCI) presented position effect (p < 0.05), because both groups presented a reduction in their values in the transition from the supine to orthostatic position (Figure 1).

Regarding the symbolic analysis indexes, there was an effect of both groups and position on 0V%, 1V% and 2LV%. The active postural maneuver led to an increase on 0V% and a decrease on 1V% and 2LV% for both groups. In addition, T2D presented higher values for 0V% and lower for the index 1V% and 2LV% in both positions when compared with CG. Whereas the index 2UV% was affected only by position effect, which shows that regardless of the group, there is alteration of this index with active postural change (Figure 1).

Table 3 show that there was not a correlation [28] between parameters of HRV and HbA1c, duration of diabetes and indexes of slow deep breathing in T2D.

**Table 3:** Correlation between the parameters of heart rate variability and glycated hemoglobin, duration of diabetes and indexes of slow deep breathing in T2D

	**0V%**	**1V%**	**2LV%**	**2UV%**	**SE**	**NCI**
Supine						
HbA1c (%)	r = −0.03	r = −0.07	r = −0.10	r = 0.20	r = 0.01	r = − 0.03
	*p* = 0.91	*p* = −0.78	*p* = 0.67	*p =* 0.40	*p =* 0.94	*p =* 0.88
Duration of Diabetes	r = 0.02	r = 0.17	r = 0.30	r = −0.25	r = 0.12	r = −0.19
	*p* = 0.93	*p* = 0.50	*p* = 0.22	*p* = 0.29	*p* = 0.63	*p* = 0.43
E/I	r = −0.24	r = 0.35	r = 0.13	r = 0.18	r = 0.16	r = 0.31
	*p* = 0.35	*p* = 0.18	*p* = 0.63	*p =* 0.50	*p =* 0.55	*p =* 0.23
ΔIE	r = 0.08	r = 0.256	r = − 0.19	r = − 0.03	r = − 0.24	r = 0.21
	*p* = 0.78	*p* = 0.34	*p* = 0.50	*p =* 0.93	*p =* 0.37	*p =* 0.44
Orthostatic						
HbA1c (%)	*r = −0.13*	*r = −0.06*	*r = −0.08*	*r = −0.08*	*r = −*0.01	*r = 0.02*
	*p =* 0.50	*p = 0.60*	*p = 0.80*	*p = 0.71*	*p = 0.94*	*p = 0.91*
Duration of Diabetes	*r = 0.17*	*r = −0.01*	*r = −0.19*	*r = −0.07*	*r = −*0.10	*r = −0.35*
	*p =* 0.50	*p =* 0.96	*p =* 0.42	*p =* 0.76	*p =* 0.67	*p =* 0.14
E/I	r = −0.16	r = 0.22	r = − 0.10	r = 0.13	r = 0.01	r = 0.14
	*p* = 0.56	*p* = 0.42	*p* = 0.71	*p =* 0.63	*p =* 0.97	*p =* 0.61
ΔIE	r = 0.23	r = − 0.274	r = − 0.33	r = 0.14	r = − 0.34	r = 0.18
	*p* = 0.39	*p* = 0.30	*p* = 0.22	*p =* 0.62	*p =* 0.20	*p =* 0.52

T2D = group type 2 diabetes; Symbolic analysis indexes (0V%, 1V%, 2LV% and 2UV%); SE = Shannon Entropy; CI = complexity Index; NCI = normalized complexity index; E/I = relationship between expiration and inspiration; ΔIE = heart rate during inspiration - heart rate during expiration. There were no significant correlations.

## Discussion

The main finding of the present study is that individuals with type 2 diabetes without diagnosed of CAN presented higher sympathetic modulation than CG, but the imbalance of the autonomic modulation did not influence the complexity of HRV yet. In addition, the response of ANS in the heart remains preserved after active postural maneuver in individuals with type 2 diabetes without CAN.

### Effect of diabetes

In the present study we observed that T2D presented an increase of sympathetic modulation, characterized by an increase of index 0V% regardless of the position adopted. In addition, T2D presented decrease of 2LV% that reflects sympathetic and parasympathetic modulation, with parasympathetic predominance. There was not difference between groups for 2UV% that reflects exclusively vagal modulation. Javorka et al. [9] also found a reduction in 2LV% with statistically significant difference when compared youngsters with type 1 diabetes with the control group in the supine position.

In addition, T2D presented a lower 1V% value than CG, in spite of its position. Probably, this result is due to the dysfunction of the ANS because this index reflects both sympathetic and parasympathetic modulation, with sympathetic predominance [25].

In the individuals with type 2 diabetes who develop CAN, the first to present lesion is the vagal nerve, and this allows a greater action of the sympathetic system on the heart [29]. However, our subjects did not present CAN, perhaps, they did not present irreversible lesion in vagal nerve yet, because there was not any difference between T2D and CG for 2UV%, but T2D presented a autonomic dysfunction reflected in a decrease of 2LV% and an increase of 0V%. Javorka et al. [30] suggested that the autonomic dysfunction in initial phases may be a consequence of functional changes of the neurons which can be reversible, for example mentioned by the authors, endoneural edema caused by hyperglycemia.

In the study of Khandoker et al. [31], it was observed that patients with type 2 diabetes with CAN presented lower complexity evaluated by sample entropy in comparison with the group with type 2 diabetes without neuropathy. Javorka et al. [30] did not find difference between healthy subjects and subjects with type 1 diabetes for complexity of HRV using also sample entropy. In another study, using NCI, the authors observed a reduction of complexity in subjects with type 1 diabetes compared with healthy subjects [9]. In our study, we did not find difference between subjects with type 2 diabetes and healthy subjects for complexity of HRV evaluated by Shannon entropy (SE) (that quantifies the degrees of complexity of the distribution of temporal sequence patterns of HRV that may be absent or not very frequent, determining lower complexity) and conditional entropy (NCI) (form in which these patterns are related, that is to say, the sequence in which they are organized). Nevertheless, it is interesting to note that in the present study, the complexity of HRV was not affected by the presence of diabetes, this is possibly due to the absence of cardiac autonomic neuropathy installed, even with known autonomic imbalance.

### Effect of postural change

Active postural maneuver is an important method to evaluate the HRV because it stimulates the ANS, and moreover it is a simpler and cheaper than tilt test [16,18]. In the present study both groups presented an increased in the heart rate (HR) and a reduction in the mean of RRi with active postural maneuver. A similar result was observed by Perseguini et al. [16] with postural change in healthy men.

Indexes of symbolic analysis showed effect of postural maneuver in modulation of ANS in heart for both groups. There was increasing 0V% and reduced 1V%, 2LV% and 2UV%. Similar results were found in other studies [14,25,32]. This response may be attributed to the reduction in venous return, and consequently, an elevation in HR, due to cardiac autonomic regulation (inhibition of vagal and stimulation of sympathetic modulation), which is mediated by the adjustments of the cardiopulmonary and arterial baroreceptors with postural change from the supine to the orthostatic position [33]. These results showed that both groups responded to maneuver, i.e., the autonomic modulation of T2D without CAN remains able to properly respond to active postural maneuver.

As regards Shannon entropy analysis, both groups did not present a reduction in complexity with postural change. These findings were not in agreement with those of the study of Porta et al. [25], who evaluated healthy subjects and observed a reduction in Shannon entropy during the tilt test, and attributed this response to the increase in the percentage of absent patterns. This difference may be due to postural change have been active and not through a postural table, ie the active postural maneuvers may have less influence in ANS compared to tilt test.

Conditional entropy is a measure of complexity of the dynamics between a pattern and the next one (regularity), so that the higher the regularity, the lower the value of conditional entropy index (NCI) [25]. Porta et al. [34], studying healthy young subjects, observed an increase in the regularity of the temporal sequence with the tilt test, and attributed this finding to the increase in sympathetic modulation and reduction in parasympathetic modulation, which were capable of reducing the complexity of the RRi. In the present study NCI also presented reduction with active postural change in both groups, which shows a greater regularity in the temporal sequence with orthostatic change, due to sympathetic predominance (0V%) and reduction in parasympathetic modulation (2UV%) in response to the active postural maneuver. These results show that there is a reduction in complexity in the presence of an increase in sympathetic modulation and decrease in vagal modulation. This data is in accordance with Porta et al. [26]. Therefore, both groups had reduction in complexity (NCI) with active postural change. Thus, these groups presented a preserved response of cardiac autonomic modulation after active postural maneuver, i.e., diabetes did not influence the results of complexity evaluated with active postural maneuvers. We attributed this result to the absence of CAN, since the subjects with diabetes did not have neuropathy installed.

### Glycemic control and duration of diabetes

The injury mechanisms of sympathetic and parasympathetic branches are not completely understood. Several hypotheses are considered in the process of pathogenesis of CAN. The hyperglycemia is considered the permissive pathogenetic factor [35] because it activates various biochemical pathway leading development and progression of CAN [5,36,37]. Therefore, due to this hypothesis, glycemic control is considered the main approach in the treatment of CAN [35]. Furthermore, the duration of the disease has been studied as a factor involved in cardiovascular autonomic neuropathy [38].

Previous studies with diabetes type 1 confirmed the correlation between HRV parameters and HbA1c and duration of diabetes [39,40], but there are others studies that did not confirm these association [30,38]. Study with diabetes type 1 and 2 found no association between HRV and HbA1c [38]. While Nolan et al. [41] found a negative association of vagal modulation with the duration of type 2 diabetes in men. In the present study there was not any association between HRV and HbA1c and duration of type 2 diabetes.

### Clinical implications

The use of the symbolic analysis in the periodic evaluation of subjects with type 2 diabetes may help in the early diagnosis of cardiac autonomic imbalance and even CAN, which allows interventions and guidance to be provided, before greater complications become established [5].

Subjects with type 2 diabetes without cardiovascular autonomic neuropathy installed may have cardiac autonomic dysfunction. It can be observed by the fact that T2D began with increased value of the index related to the sympathetic autonomic system (0V%) and decreased value of the 2LV% that reflects sympathetic and parasympathetic with predominance vagal modulation. The evaluation of HRV before and after active postural maneuver is a simple and low cost test that can be performed at the bedside [16]. Furthermore, this test may be used in the clinical evaluation because it is a stimulus for ANS [16,18]. However, in present investigation, it was not possible to observe differences between groups with this test. This, possibly, occurred due to lack of neuropathy in TD2 and both groups present ANS response with active postural maneuvers.

### Study limitations

**Figure 2:**
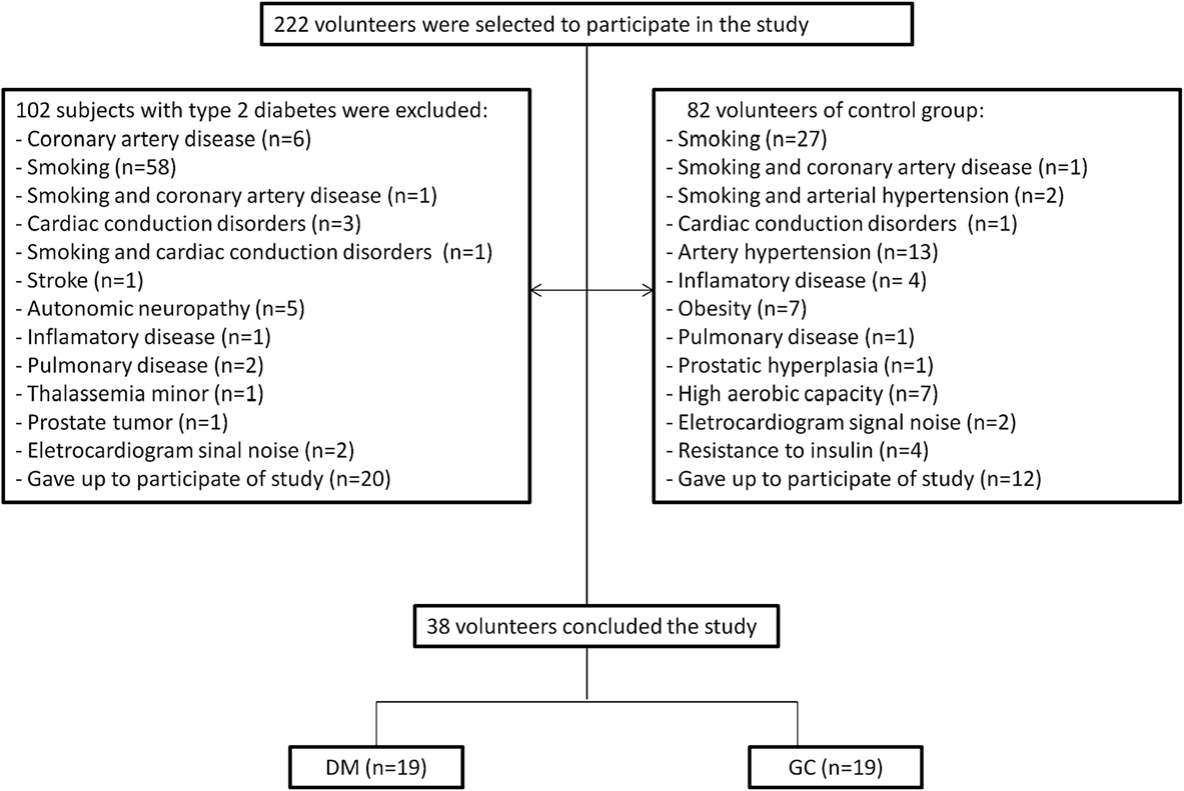
Flow diagram indicating sampling loss in the study.

The number of subjects studied in each group is small due to the sample loss during the study (Figure 2). However, it is noteworthy that the number of subjects was sufficient to detect some important differences in HRV as described above. Furthermore, we did not analyze the fasting glucose and insulin (laboratory tests that are common in the literature), however there are studies that did not show correlation between these laboratory tests and HRV [42,43].

## Conclusions

The present study showed that individuals with type 2 diabetes without CAN presented higher sympathetic modulation. However, the complexity of HRV was not influenced by imbalance of the autonomic modulation in individuals with type 2 diabetes. In addition, the response of ANS in the heart remains preserved after active postural maneuver in individuals with type 2 diabetes, possibly due to lack of CAN in this group.

## Abbreviations

ANS: Autonomic nervous system; BMI: Body mass index; CAN: Cardiovascular autonomic neuropathy; CG: Control group; CRP: C-reactive protein; ΔIE: Difference between heart beat during inspiration and expiration; E/I: Ratio of the longest RR interval during expiration and the shortest RR interval during inspiration; HbA1c: Glycated hemoglobin; HR: Heart rate; HRV: Heart rate variability; NCI: Normalized complexity index; RRi: Interval between successive R waves; SE: Shannon entropy; T2D: Group of individuals with type 2 diabetes; VO2peak: Maximum volume of oxygen consumption.

## Competing interests

The authors declare that they have no competing interests.

## Authors’ contributions

SCGMT: Participated in the conception and design of the study, acquisition and analysis of data, interpretation of data, performed the statistical analysis and drafted the manuscript. ACMT: Participated in the analysis and interpretation of data helped to draft the manuscript. COF: Participated in the conception and design of the study, collection, acquisition of data and helped to draft the manuscript. SLBL: Participated in cardiology exam, clinical ergometric test and acquisition of data and revising it critically for important intellectual content. AMDV: Participated in collection and acquisition of data and revising it critically of manuscript. ABS: Participated in acquisition of data and revising it critically of manuscript. AMOL: Participated in the conception and design of the study and revising it critically of manuscript. NM: Participated in the analysis and interpretation of data and revising it critically for important intellectual content. AP: Participated in the analysis and interpretation of data and revising it critically for important intellectual content. AMC: Participated in the conception, design and coordination of the study and revising it critically for important intellectual content. All authors read and approved the final manuscript.

## Authors’ information

Sílvia Cristina Garcia de Moura Tonello is master in physiotherapy from Federal University of São Carlos, research interests and experiences on Diabetes Mellitus, cardiorespiratory fitness, pulmonary function and heart rate variability.

Anielle Cristhine de Medeiros Takahashi is professor in the Department of Physiotherapy in Federal University of São Carlos, São Paulo, Brazil. Her research interests are aging, frailty, complexity of biological signals.

Cristina O. Francisco is master in physiotherapy from Federal University of São Carlos, research interests and experiences on Diabetes Mellitus, cardiorespiratory fitness, pulmonary function and inflammation.

Sérgio Luiz Brasileiro Lopes is professor in the Department of Medicine, Federal University of São Carlos, specialized by the Brazilian Medical Association in Clinical Medicine, Cardiology and Intensive Care.

Adriano M. Del Vale is physician specialized in pathology. Collaborator of Cardiovascular Physiotherapy Laboratory, Nucleus of Research in Physical Exercise at Physiotherapy Department at Federal University of São Carlos, São Paulo, Brazil.

Audrey Borghi e Silva is professor in the Department of Physiotherapy in Federal University of São Carlos, São Paulo, Brazil. She has experience in the area of physical therapy, with emphasis on Cardiorespiratory Physiotherapy and Exercise Physiology. Her research interests are non-invasive ventilation, ergogenic supplementation and study of pathophysiological mechanisms of exercise intolerance in cardiorespiratory disorders chronic.

Ângela Merice de Oliveira Leal is professor in the Department of Medicine in Federal University of São Carlos. She has experience in the area of Medicine, with emphasis in Clinical Medicine, Endocrinology and Metabolism. Her research interests are endocrinology, diabetes and metabolism, molecular biology and system activin-myostatin.

Nicola Montano is professor in Department of Clinical Sciences, Internal Medicine II, L. Sacco Hospital, University of Milan, Milan, Italy. His research interests are neural control of cardiovascular function and the relationship between neural and cardiovascular oscillatory patterns.

Alberto Porta is professor in Department of Technologies for Health, Galeazzi Orthopaedic Institute, University of Milan, Milan, Italy. His research interests are time series analysis, nonlinear dynamics, system identification, and modeling applied to cardiovascular control mechanisms.

Aparecida Maria Catai is professor in the Department of Physiotherapy in Federal University of São Carlos, São Paulo, Brazil. She has experience in the area of physical therapy with emphasis in cardiovascular physical therapy and exercise physiology, responses of heart rate, blood pressure, heart rate variability, blood pressure variability, anaerobic threshold and ventilatory limitation to exercise, aerobic and resistive exercise and aging, cardiovascular diseases, respiratory and metabolic.
